# A novel m6A‐related prognostic signature for predicting the overall survival of hepatocellular carcinoma patients

**DOI:** 10.1049/syb2.12036

**Published:** 2021-10-14

**Authors:** Shiyang Xie, Yaxuan Wang, Jin Huang, Guang Li

**Affiliations:** ^1^ Department of Radiation Oncology The First Hospital of China Medical University Shenyang China; ^2^ Department of Anesthesiology The First Hospital of China Medical University Shenyang China

**Keywords:** gene signature, hepatocellular carcinoma, m6A, prognosis, TCGA

## Abstract

Liver hepatocellular carcinoma (LIHC) comprises most cases of liver cancer with a poor prognosis. *N* ^6^‐methyladenosine (m6A) plays important biological functions in cancers. Thus, the present research was aimed to determine biomarkers of m6A regulators that could effectively predict the prognosis of LIHC patients. Based on the data collected from the Cancer Genome Atlas (TCGA) database, the correlation between the mRNA expression levels and copy number variation (CNV) patterns were determined. Higher mRNA expression resulted from the increasing number of 9 genes. Using the univariate Cox regression analysis, 11 m6A regulators that had close correlations with the LIHC prognosis were identified. In addition, under the support of the multivariate Cox regression models and the least absolute shrinkage and selection operator, a 4‐gene (YTHDF2, IGF2BP3, KIAA1429, and ALKBH5) signature of m6A regulators was constructed. This signature was expected to present a prognostic value in LIHC (log‐rank test *p* value < 0.0001). The GSE76427 (*n* = 94) and ICGC‐LIRI‐JP (*n* = 212) datasets were used to validate the prognostic signature, suggesting strong power to predict patients' prognosis for LIHC. To sum up, genetic alterations in m6A regulatory genes were identified as reliable and effective biomarkers for predicting the prognosis of LIHC patients.

## 1 INTRODUCTION

In 2018, liver cancer was ranked the fourth major reason for cancer‐related deaths and the sixth most common cancer worldwide, leading to nearly 782,000 deaths and 841,000 new cases [1]. Among all liver cancer cases, liver hepatocellular carcinoma (LIHC) comprises 75%–85% of the cases [1]. Hepatitis B virus (HBV) is one of the main risk factors for LIHC, which often results in cirrhosis [2]. Thus, since 1982, the primary prevention of most LIHC cases has been feasible through vaccines against HBV. With the rapid development of medical technology, the diagnosis and treatment of LIHC have greatly improved, but its prognosis is still very poor. In 2010, the estimated number of deaths in China from LIHC was 312,432 [3]. In addition, in the United States, during the period 2004–2009, the 5‐year survival rate of the localised‐stage disease was 25.7%, the regional‐stage disease was 9.5%, and the distant‐stage disease was 3.5% [4]. Therefore, finding reliable and effective biomarkers for predicting the prognosis of LIHC patients becomes highly necessary.

*N* ^6^‐methyladenosine (m6A) is the most common RNA methylation, which occurs in approximately 25% of transcripts at the genome‐wide level [5]. This modification can regulate RNA splicing, degradation, and translation into proteins that are catalysed by RNA methyltransferases and called ‘writers’, including METTL3, METTL14, and METTL16. The ‘eraser’ genes include FTO and ALKBH5, and the ‘reader’ genes interact with m6A‐binding proteins, such as YTHDF1 and IGF2BP1. Previous research has demonstrated the significant biological functions of m6A in a wide range of human cancers, including proliferation, differentiation, and tumourigenesis [6, 7, 8]. For example, Li et al. [9] determined that FTO can have a carcinogenic effect on m6A demethylase in acute myeloid leukaemia. Another study proved that FTO played a crucial role as an m6A demethylase, which enhanced anti‐PD‐1 resistance and melanoma tumourigenesis [10]. In glioma, the relationship between 24 functional single‐nucleotide polymorphisms (SNPs) in eight m6A modification core genes and the risk were determined [11]. As one of the ‘writers’ gene, WTAP SNPs were proved to be genetic modifiers for the development of hepatoblastoma in Chinese children [12]. Another ‘writers’ gene was named METTL14. Zhuo et al. [13] found that some SNPs in the METTL14 gene are associated with predisposition to neuroblastoma in children.

Because of available gene expression databases, such as the Cancer Genome Atlas (TCGA) [14], people have recently begun to understand the genetic changes in human malignant tumours more intuitively. Based on the TCGA, Zhou et al. [15] reported genetic alterations in m6A regulators in clear cell renal cell carcinoma and identified how these alterations correlated with clinical characteristics. In addition, they identified m6A RNA methylation regulators to be important players in the malignant progression of glioma, which may be useful for the development of prognostic stratification and therapeutic strategies [16].

In the present study based on TCGA data, a comprehensive bioinformatic analysis of gene signatures was performed, together with an evaluation of prognostic values of m6A regulators in LIHC. Information on LIHC patients, including clinical data as well as the single nucleotide variant (SNV), copy number variation (CNV), and gene expression, were obtained. Significant correlations were found between CNV patterns and the mRNA expression levels of m6A regulators. Through the univariate Cox regression analysis, we found 11 m6A regulators in total that had a great correlation with LIHC prognosis. Under the support of the least absolute shrinkage and selection operator (LASSO) and multivariate Cox regression models, a 4‐gene signature of m6A regulators was constructed, showing the value in the LIHC prognosis. It is able to achieve an effective prediction of the LIHC prognosis. According to the pathway enrichment analysis, high YTHDF2 expression is associated with DNA repair, DNA recombination, and exonuclease activity. In addition, high IGF2BP3 expression is associated with double‐strand break repair and ATP‐dependent chromatin remodelling. To sum up, genetic alterations in m6A regulatory genes were determined as reliable and effective biomarkers for predicting the prognosis of LIHC patients.

## 2 MATERIALS AND METHODS

### 2.1 Data resource and processing

In this study, all clinical information on LIHC patients, such as mRNA expression data, SNV, CNV etc., was retrieved from the TCGA database [14] using the TCGA‐assembler software [17] in June 2019. For the mRNA expression data, we obtained 423 LIHC samples and downloaded the data as TPM files. With regard to the SNV data, 364 samples in total were collected, and the data downloaded were level 3 processed with MuTect [18]. In addition, there were 766 samples for the CNV data. Deletions refer to a Segment_Mean < −0.2, and amplifications refer to a Segment_Mean > 0.2. Regarding clinical information, there were 377 LIHC samples with survival and clinicopathological data. Finally, after integrating all data, a total of 321 samples were available for further analysis after the samples that had survival time no more than 90 days or lacked some clinical information were excluded.

### 2.2 LASSO model

The LASSO model, which draws on the penalty method, can be used to select the variables of the sample data. It directly compresses the original small coefficients to 0. Thus, the corresponding variables are regarded as insignificant. The glmnet package in R [19] was employed to generate this model.

### 2.3 Validation of the prognostic signature of the m6A regulators

To validate the constructed prognostic signature, RNA‐seq data from the International Cancer Genome Consortium (ICGC) database (https://icgc.org/) and gene microarray data from the Gene Expression Omnibus (GEO) database (https://www.ncbi.nlm.nih.gov/geo/) [20] were used. Here, the GSE76427 dataset [21] contained 94 LIHC patients. The ICGC‐LIRI‐JP project from the ICGA database contained 212 LIHC patients. Both of these two datasets had clinical information matching with gene expression data. Using the gene expression data of m6A regulators from our signature, we performed the validation analysis by the cut off of the median risk score.

### 2.4 Gene set enrichment analysis (GSEA)

We conducted GSEA using the software that was freely available on the website (http://software.broadinstitute.org) [22] and classified all samples into low‐ and high‐expression sets by the median expression level. In terms of the significantly enriched gene sets, the false discovery rate and the normalised *p* value should be less than 0.25 and 0.05, respectively.

### 2.5 Cell line and cell culture

The human hepatocellular carcinoma cell line HepG2 was purchased from the Cell Bank of Type Culture Collection of Chinese Academy of Sciences, Shanghai Institute of Cell Biology, Chinese Academy of Sciences. These cell lines were cultured in RPMI‐1640 (Hyclone, LA, USA) supplemented with 10% foetal bovine serum (FBS) (Clark, USA) in a humidified incubator containing 5% CO_2_.

### 2.6 Construction of shRNA expression lentivirus

For RNA interference, four short hairpin RNA (shRNA) targets of IGF2BP3 were designed and separately cloned into a pGFP‐puro‐IGF2BP3 lentiviral vector. The infectious lentivirus was produced by transfecting the lentivector and packaging vectors into 293T cells. The puromycin resistance gene (pac) and luciferase full‐length cDNA were cloned into the expressing construct and used for the lentivirus package. In addition, a negative control plasmid with scrambled shRNA and a positive pac shRNA plasmid were constructed. All shRNA plasmids were synthesised by GenePharma (Shanghai, China).

### 2.7 Transfection and isolation of stable cell clones

To generate HCC cell lines with IGF2BP3 knockdown, lentiviral pGFP271‐puro‐IGF2BP3 shRNA and control lentiviral pGLV3‐puro‐control shRNA were used to infect the HepG2 cell line according to the manufacturer's instruction. The efficiency of transfection was assessed by fluorescence microscopy. Cells were selected using puromycin for 2 weeks, and HCC cell lines with a stable knockdown of IGF2BP3 and the control cell line with an empty vector were obtained.

### 2.8 Irradiation condition

The HCC cells were irradiated using a 6‐MV X‐ray linear accelerator (model: Varian Medical System, CA, USA) at a dose rate of 300 cGy/min. The radiation doses were 0, 2, 4, 6, AND 8 Gy, respectively. The cells were placed in the incubator and samples were collected at the indicated time points (0, 1, 12, 24, 48 h).

### 2.9 Cell proliferation assay

Cell viability of IGF2BP3 knockdown cells were determined by the methylthiazol tetrazolium (MTT) assay. The cells (2000 cells per well) were seeded in 96‐well plates. After the treatment of different radiation doses (0, 2, 4, 6, 8 Gy), the cells were incubated for another 24 h. Each radiation dose was applied to 3 times the wells of cells. The cell viability assay was performed using with MTT Cell Proliferation and Cytotoxicity Assay Kit (Beyotime, China) according to manufacturer's instructions. 10 μL of MTT (5 mg/ml) was added to each well. The cells were incubated at 37°C for an additional 4 h. At the end of the incubation, 100 μL of formazan solution was added to each well. The absorbance of each well was monitored using a spectrophotometer at 570 nm (A570). The experiments were performed in triplicate.

### 2.10 Colony formation assay

Cells were seeded in triplicate in 6‐well plates at a density of 1 × 103 cells per well. Following overnight attachment, the plates were exposed to 4 Gy of IR. After incubation for 12 days, colonies were washed twice with PBS, fixed with methanol and stained with 0.5% crystal violet. Colonies containing more than 50 cells were counted as surviving clones. The experiments were performed in triplicate.

### 2.11 Wound‐healing assay

Cells were seeded in 6‐well plates and grew to confluence. The plates were washed twice with PBS, and a serum‐free medium was added to the plates. Three parallel ‘wounding’ lines were scratched into the cell monolayer with a sterile 1000‐μL pipette tip separately in each plate. The width of the wound area was photographed and measured under the inverted phase contrast microscope (40X magnification, Nikon, Japan) to assess cell migration at 0, 12, 24 and 48 h after scratching.

### 2.12 Cell migration and invasion assay

For transwell migration assays, 200 μL of 2 × 104 infected cells were plated into each top chamber using an 8‐mm pore filter insert (Corning, NY, USA). For invasion assays, 200 μL of 2 × 104 cells were plated into each top chamber of the insert covered with Matrigel in both assays, cells were plated in a medium without serum and a 500 μL serum‐containing medium (20% FBS) was used in the lower chamber as a chemoattractant. After 48 h of incubation, cells on the upper surface of the filter were removed using a cotton swab and cells that had invaded through the bottom surface of the filter were fixed with methanol and stained with crystal violet, imaged and counted in 10 randomly selected viewing fields under the microscope (100X magnification).

### 2.13 Statistical analysis

The analysis of all statistical data was in R language. How the prognosis was correlated with clinicopathological characteristics, CNVs, and SNVs in m6A regulatory genes was explored through the univariate Cox regression analysis. The chi‐square test was conducted for examining how CNVs and SNVs in m6A regulatory genes correlated with alternation in LIHC key genes. The comparisons between two independent groups of samples and between multiple independent samples were performed with the Wilcoxon test and the Kruskal–Wallis test, respectively. The prognosis of the two risk groups was compared by a Kaplan–Meier curve, with differences evaluated by the log‐rank test. The sensitivity and specificity in predicting overall survival were analysed by a ROC curve, with the area under the ROC curve (AUC) value. A *p* value < 0.05 was identified as significant.

## 3 RESULTS

### 3.1 SNVs and CNVs in m6A regulatory genes in LIHC patients

**FIGURE 1:**
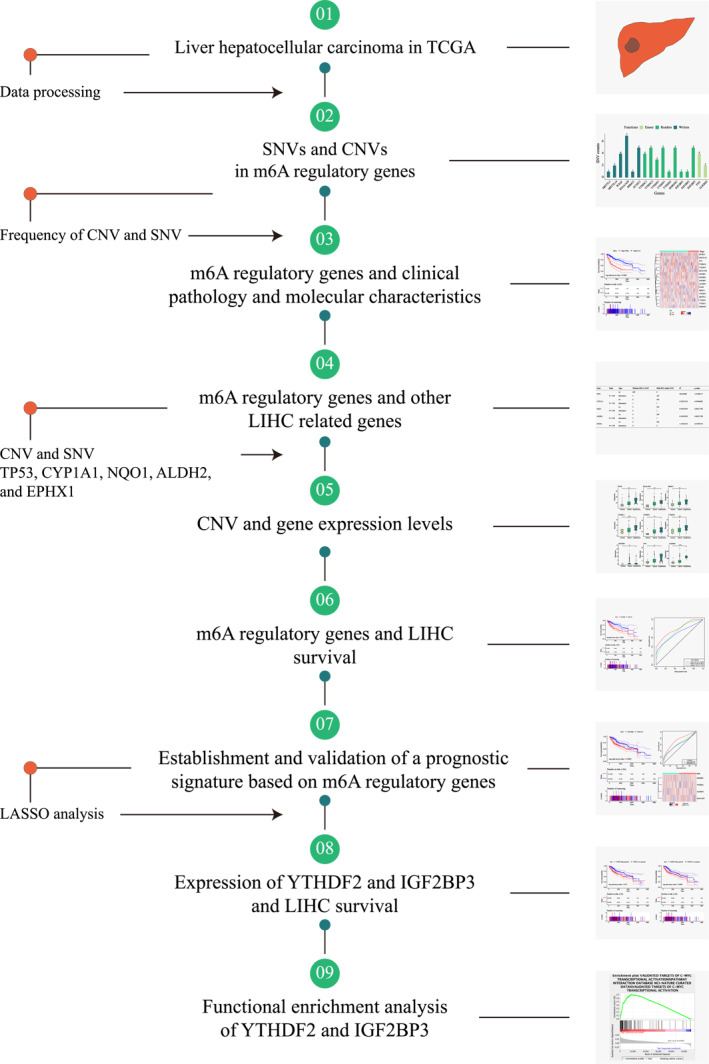
Research workflow. Gene signatures and prognostic values of *N* ^6^‐methyladenosine regulators in liver hepatocellular carcinoma based on the Cancer Genome Atlas database

**FIGURE 2:**
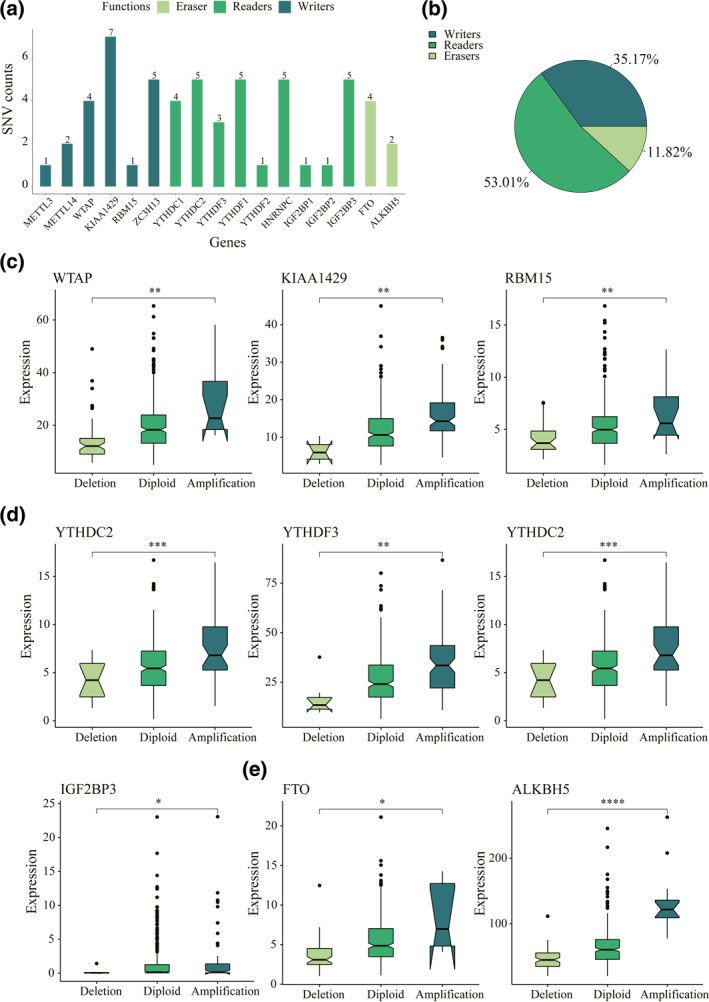
Single nucleotide variants (SNVs), copy number variations (CNVs) and gene expression of *N* ^6^‐methyladenosine (m6A) regulatory genes in liver hepatocellular carcinoma (LIHC) patients. (a) Frequency of mutations in different m6A regulatory genes in LIHC samples. (b) The CNV statistics of m6A regulatory genes in LIHC samples. (c) The CNVs and expression levels of writer genes. (d) The CNVs and expression levels of reader genes. (e) The CNVs and expression levels of eraser genes. *p value < 0.05, **p value < 0.01, ***p value < 0.001 and ****p value ≤ 0.0001

Herein, a complete bioinformatic analysis was performed to find out gene signatures and prognostic values of m6A regulators in LIHC with TCGA data (Figure 1). A total of 17 m6A regulatory genes were selected. The mutations in m6A regulatory genes were found from 57 independent samples among the SNV data of 364 patients (Table 1). The highest mutation was located in the ‘writer’ gene KIAA1429 of six LIHC samples. The ‘eraser’ genes had a higher mutation frequency overall; it, together with the ‘writer’ genes, had a lower frequency than the ‘reader’ genes (Figure 2a). Moreover, among the 766 LIHC samples containing CNV data, a high frequency of CNVs was found in the m6A regulatory genes (Figure 2b). The highest frequency of CNV events at 28.94% came from the ‘writer’ gene KIAA1429, followed by the second ‘reader’ gene YTHDF3 at 27.45% (Table 2). Furthermore, among all CNVs in m6A regulatory genes, the most frequent alteration was observed in a copy number gain of KIAA1429, and deletions in ZC3H13 ranked first among all CNVs.

**TABLE 1:** Mutations in m6A regulatory genes in top 10 LIHC patients

	Writers						Readers									Erasers	
Samples	METTL3	METTL14	WTAP	KIAA1429	RBM15	ZC3H13	YTHDC1	YTHDC2	YTHDF3	YTHDF1	YTHDF2	HNRNPC	IGF2BP1	IGF2BP2	IGF2BP3	FTO	ALKBH5
TCGA‐2Y‐A9H8																5′UTR	
TCGA‐2Y‐A9H9							p.F492L		p.P512P								
TCGA‐4R‐AA8I										p.D401G							
TCGA‐5R‐AA1C				p.Q68*													
TCGA‐BC‐A10Y									p.G193V								
TCGA‐BC‐A216			intron										p.X273_splice				
TCGA‐BD‐A3ER						p.S19N											
TCGA‐CC‐5264										p.H24N, p.L23Ffs*5							
TCGA‐CC‐A3M9								p.M795T									

Abbreviations: LIHC, Liver hepatocellular carcinoma; m6A, *N* ^6^‐methyladenosine; TCGA, The Cancer Genome Atlas.

^*^represents the position of the last amino acid of the frameshift mutation.

**TABLE 2:** The CNV statistics of m6A regulatory genes in LIHC samples

Type	Gene	Diploid	Deletion	Amplification	CNVs	Deletion %	Amplification %	Percentage
Writers	METTL3	694	41	31	72	56.94	43.06	9.40
	METTL14	626	132	8	140	94.29	5.71	18.28
	WTAP	618	125	25	150	83.33	16.67	19.53
	KIAA1429	545	10	212	222	4.50	95.50	28.94
	RBM15	664	65	37	102	63.73	36.27	13.32
	ZC3H13	597	163	11	174	93.68	6.32	22.57
Readers	YTHDC1	644	121	14	135	89.63	10.37	17.33
	YTHDC2	655	24	87	111	21.62	78.38	14.49
	YTHDF3	563	29	184	213	13.62	86.38	27.45
	YTHDF1	663	5	98	103	4.85	95.15	13.45
	YTHDF2	642	114	10	124	91.94	8.06	16.19
	HNRNPC	697	43	37	80	53.75	46.25	10.30
	IGF2BP1	668	7	95	102	6.86	93.14	13.25
	IGF2BP2	702	18	48	66	27.27	72.73	8.59
	IGF2BP3	651	11	109	120	9.17	90.83	15.56
Erasers	FTO	630	135	16	151	89.40	10.60	19.33
	ALKBH5	591	146	30	176	82.95	17.05	22.95
Total		10,850	1189	1052	2241	53.06	46.94	17.12

Abbreviations: CNV, copy number variation; LIHC, Liver hepatocellular carcinoma; m6A, *N* ^6^‐methyladenosine.

### 3.2 CNV and SNV occurrences in m6A regulatory genes associated with clinical pathology and molecular characteristics

Next, how SNV and CNV occurrences among m6A regulatory genes correlated, as well as LIHC patients' clinicopathological features, was evaluated. First, a univariate Cox regression analysis was conducted on each clinical feature, where the tumour stage showed a significant association with LIHC patients' survival time (*p* value < 0.0001, Table S1). Although these changes in m6A regulatory genes (CNVs, CNVs or SNVs) were not significantly associated with the prognosis of LIHC, they both had negative impacts on patient survival with a hazard ratio (HR) > 1. Interestingly, the HR of SNV was <1. From the perspective of overall changes in m6A regulatory genes, for either SNV or CNV alone, or both, these changes showed insignificant correlations with the prognosis of patients (*p* value > 0.05). However, we hypothesised the possible correlations between these changes in m6A regulatory genes and other therapeutic molecules in LIHC patients. Considering the crucial role of TP53 [23], CYP1A1 [24], NQO1 [25], ALDH2 [26], and EPHX1 [27] in the pathogenesis of LIHC, we then examined how CNVs and SNVs in m6A regulatory genes correlated with alternation in these five genes. Unsurprisingly, significant associations with alternation in TP53, CYP1A1, NQO1, and ALDH2 were found. Here, only three samples among 106 LIHC patients with TP53 alterations were without SNVs and CNVs (Table 3).

**TABLE 3:** Relationship between alteration of molecular characteristics and m6A regulatory genes in LIHC patients

Gene	Total	Type	Without SNV or CNV	With SNV and/or CNV	*X* ^*2*^	*p* value
TP53		wt	258	0	344.85989	5.5782 × 10^−77^
	*N* = 364	Alternation	3	103		
CYP1A1		wt	8	354	4.23233125	0.03966093
	*N* = 364	Alternation	1	1		
NQO1		wt	9	354	9.39325478	0.00217785
	*N* = 364	Alternation	0	1		
ALDH2		wt	9	354	9.39325478	0.00217785
	*N* = 364	Alternation	0	1		
EPHX1		wt	9	350	1.19126176	0.27507519
	*N* = 364	Alternation	0	5		

Abbreviations: CNV, copy number variation; LIHC, Liver hepatocellular carcinoma; m6A, *N* ^6^‐methyladenosine; SNV, single nucleotide variant.

The previous analysis revealed significantly larger CNV changes in m6A regulatory genes compared to SNV changes, as well as the influence of CNVs on the levels of gene expression. Next, how mRNA expression levels were affected by CNVs in m6A regulatory genes was examined. A significantly close correlation was found between mRNA expression levels and CNV patterns in 423 LIHC samples. Among 17 m6A regulatory genes, mRNA expression increased as the copy numbers of nine genes rose, and it decreased with the deletion (Figure 2c–e).

### 3.3 How m6A regulatory genes correlated with the prognosis in LIHC patients

**FIGURE 3:**
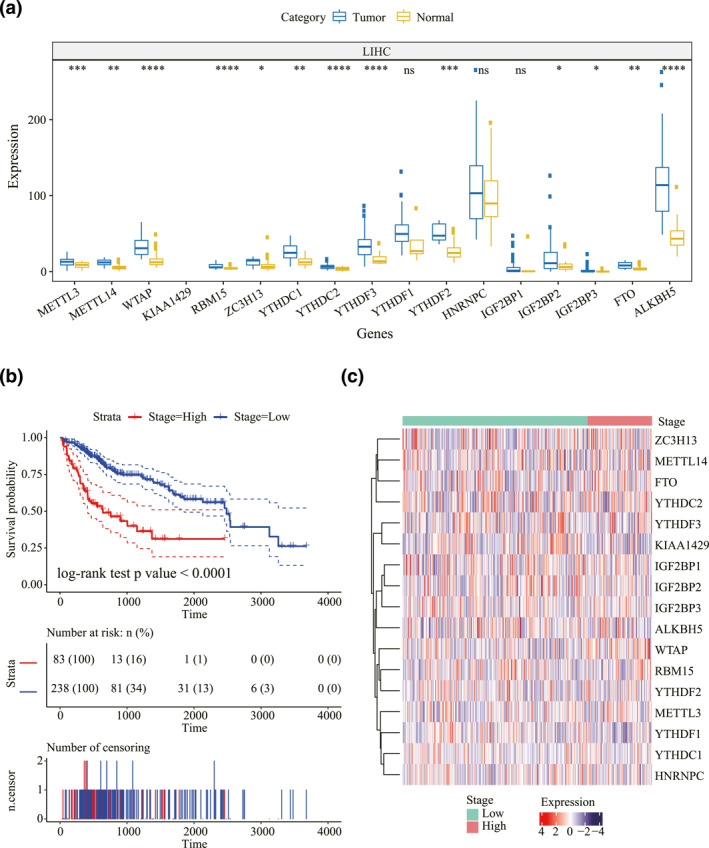
Association between expression of *N* ^6^‐methyladenosine (m6A) regulatory genes and liver hepatocellular carcinoma patients' survival. (a) The differentially expressed patterns of m6A regulator genes between tumour and normal tissues. *p value < 0.05, **p value < 0.01, ***p value < 0.001 and ****p value ≤ 0.0001. (b) Kaplan–Meier curves of tumour stages and prognosis in patients. (c) The expression levels of m6A regulatory genes in different stages

The differences in the expressed patterns between normal tissues and tumours in LIHC were observed. As shown in Figure 3a, all the m6A regulator genes except three genes (HNRNPC, IGF2BP1 and YTHDF1) were differentially expressed. A significant association of the LIHC prognosis with the tumour stage was determined (log‐rank test *p* value < 0.0001, Figure 3b) for the m6A regulatory genes' prognostic value. Here, I/II NOS, as well as stages I and II, were considered as low‐stage cases; high‐stage ones were those above Stage III. Moreover, we clustered and analysed how m6A regulatory genes were expressed in each tumour stage for LIHC (Figure 3c). The tumour stage showed a significant association with patients' survival time but not with the expression of m6A regulatory genes (Figure S1). According to the studies conducted previously, CNV in m6A regulatory genes may lead to dysregulated expression levels of genes. Afterwards, CNVs were taken as the research object to examine how CNVs in m6A regulatory genes correlated with LIHC patients' survival time. We found insignificant correlation between CNVs and patients' survival (Figure S2a), and SNVs were also not associated with the prognosis (Figure S2b).

Based on the above results, CNVs in m6A regulatory genes displayed insignificant relationships with patients' prognosis, despite a strong positive correlation of CNV changes with the expression levels of some m6A regulatory genes. Then, how the patients' prognosis correlated with m6A regulatory genes' expression was explored through univariate Cox regression analysis. As shown in Table S2, the expression of a total of 11 genes displayed a correlation with the prognosis of LIHC patients at a significant level (*p* value < 0.05). Of these 11 genes, the expression levels of five were significantly correlated with their CNV changes. How the patients' prognosis was affected by 17 m6A regulatory genes was determined by a multivariate Cox regression analysis. The expression levels of 17 m6A regulatory genes were found to be a useful factor to measure the risk of LIHC patients (Figure S3a). Notably, all the AUC values in the first year, third year and fifth year exceeded 0.7 (Figure S3b). These findings identified that a prognostic marker for LIHC patients was the expression of m6A regulatory genes.

### 3.4 Establishment and validation of a prognostic signature based on m6A regulatory genes

**FIGURE 4:**
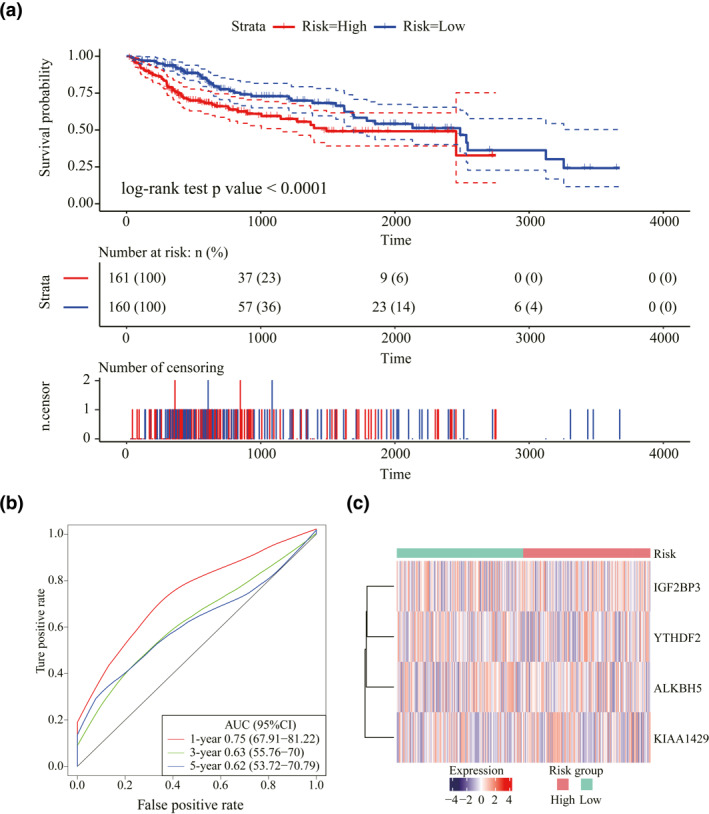
A prognostic signature constructed based on *N* ^6^‐methyladenosine regulatory genes. (a) Kaplan–Meier curves of the risk score and prognosis in liver hepatocellular carcinoma patients. (b) The ROC of the prognostic signature with 1‐year, 3‐year, and 5‐year survival. (c) Expression heatmap of four genes in the two risk groups

Drawing on the above results, we conducted a LASSO analysis on 17 m6A regulatory genes for decreasing prognostic markers. The 1000 LASSO regressions demonstrated four genes YTHDF2, KIAA1429, IGF2BP3, and ALKBH5 in the LASSO results over 100 times as well as a considerable influence of their CNVs on the expression levels (Table 4). Three major m6A regulatory functions of erasers, readers and writers are covered in these genes. Then, the risk of LIHC patients as predicted with the expression of these four genes was calculated. Specifically, a multivariate Cox regression analysis was performed on these four genes to calculate the patient's risk scores. The median risk score was taken as a cut off. Then, these four genes were found to be effective in predicting the survival time of LIHC patients. The two risk groups displayed significant differences in the prognosis (log‐rank test *p* value < 0.0001, Figure 4a). Furthermore, all the AUC values of these four genes in the first year, third year, and fifth year exceeded 0.6 (Figure 4b). Similarly, the expression levels of these four m6A regulatory genes were clustered, together with patients' risk scores. Different genes were found predisposed in high‐ and low‐risk patients (Figure 4c).

**TABLE 4:** The results of LASSO analysis based on m6A regulatory genes

Duplicates	Genes	CNV and expression	Functions
926	YTHDF2	Yes	Readers
904	IGF2BP3	Yes	Readers
442	KIAA1429	Yes	Writers
269	ALKBH5	Yes	Erasers
44	RBM15	Yes	Writers
43	WTAP	Yes	Writers
30	YTHDC2	Yes	Readers
24	FTO	Yes	Erasers
23	YTHDF3	Yes	Readers
19	YTHDC1	No	Readers
13	HNRNPC	No	Readers
985	ZC3H13	No	Writers
961	YTHDF1	No	Readers
557	METTL14	No	Writers
337	METTL3	No	Writers
228	IGF2BP2	No	Readers
72	IGF2BP1	No	Readers

Abbreviations: CNV, copy number variation; LASSO, least absolute shrinkage and selection operator; m6A, *N* ^6^‐methyladenosine.

**FIGURE 5:**
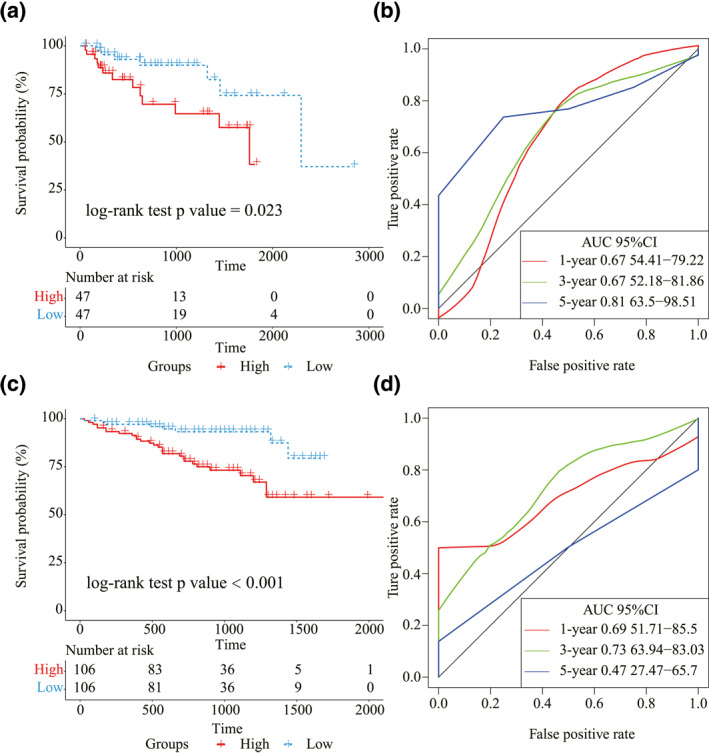
Validation of the constructed prognostic signature. (a) Kaplan–Meier curves of the risk score and prognosis in liver hepatocellular carcinoma (LIHC) patients in the Gene Expression Omnibus (GEO) dataset. (b) The ROC of the prognostic signature with 1‐year, 3‐year, and 5‐year survival in the GEO dataset. (c) Kaplan–Meier curves of the risk score and prognosis in LIHC patients in the International Cancer Genome Consortium (ICGC) dataset. (d) The ROC of the prognostic signature with 1‐year, 3‐year and 5‐year survival in the ICGC dataset

In order to validate our m6A regulators’ prognostic signature, we used GEO microarray data (GSE76427, *n* = 94) and ICGC RNA‐seq data (ICGC‐LIRI‐JP, *n* = 212). Both of these two datasets had clinical information matching with gene expression data. Each risk score of patients in the GEO and ICGC datasets was calculated with the gene expression of m6A regulators in the constructed signature. By the cut off of the median risk score, the Kaplan–Meier curves showed different survival outcomes between two risk groups in the GEO dataset (log‐rank test *p* value < 0.05, Figure 5a). All the AUC values of this signature in the first year, third year, and fifth year exceeded 0.6 (Figure 5b). In addition, there was also a significant survival difference between two risk groups in the ICGC dataset (log‐rank test *p* value < 0.001, Figure 5c). Figure 5d presents the AUC values in the first year, third year, and fifth year. The above results suggested that our four m6A regulator genes signature have strong power to predict patients' prognosis for LIHC.

### 3.5 Association between signature and survival of LIHC patients

**FIGURE 6:**
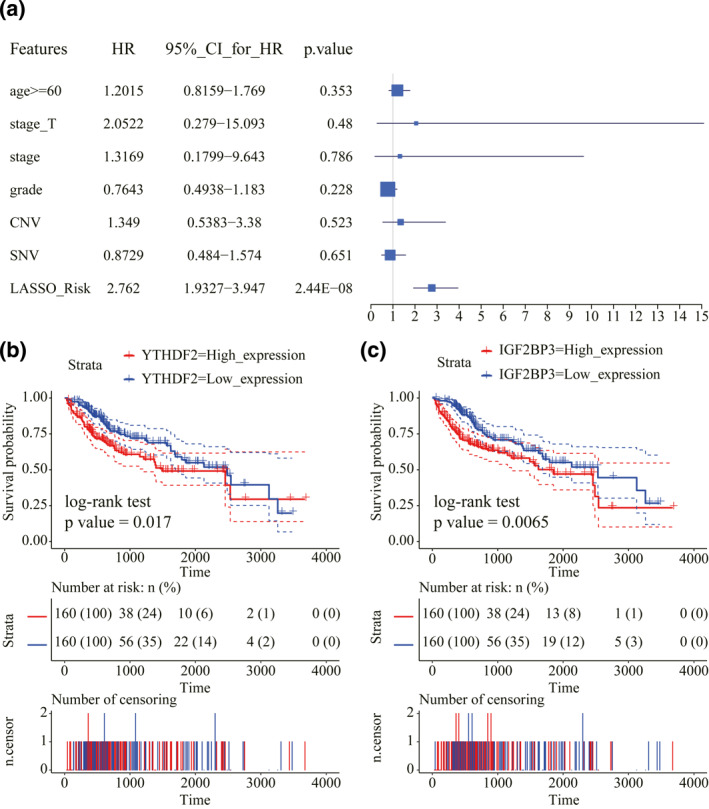
Association between signature and survival of liver hepatocellular carcinoma (LIHC) patients. (a) The risk score was confirmed by Multivariate Cox regression analysis to be an independent indicator for LIHC prognosis. (b) Kaplan–Meier curves of YTHDF2 and LIHC prognosis. (c) Kaplan–Meier curves of IGF2BP3 and LIHC prognosis

Next, clinical factors including age, T stage, tumour stage, grade, CNV, SNV and the risk score were included. The risk score was an independent prognostic indicator for OS (*p* value < 0.001, Figure 6), as found from the multivariate Cox regression analysis. We then examined the gene expression effects of the above four genes on LIHC patients' survival. The survival time of LIHC patients was found to have a significant association with the expression levels of YTHDF2 and IGF2BP3 (Figure 6b,c). Among them, patients with higher expression levels of YTHDF2 and IGF2BP3 had significantly lower survival outcomes than those with lower expression levels. It implies the important clinical implications of the expression levels of YTHDF2 and IGF2BP3 for LIHC patients.

### 3.6 Functional enrichment analysis of YTHDF2 and IGF2BP3

**FIGURE 7:**
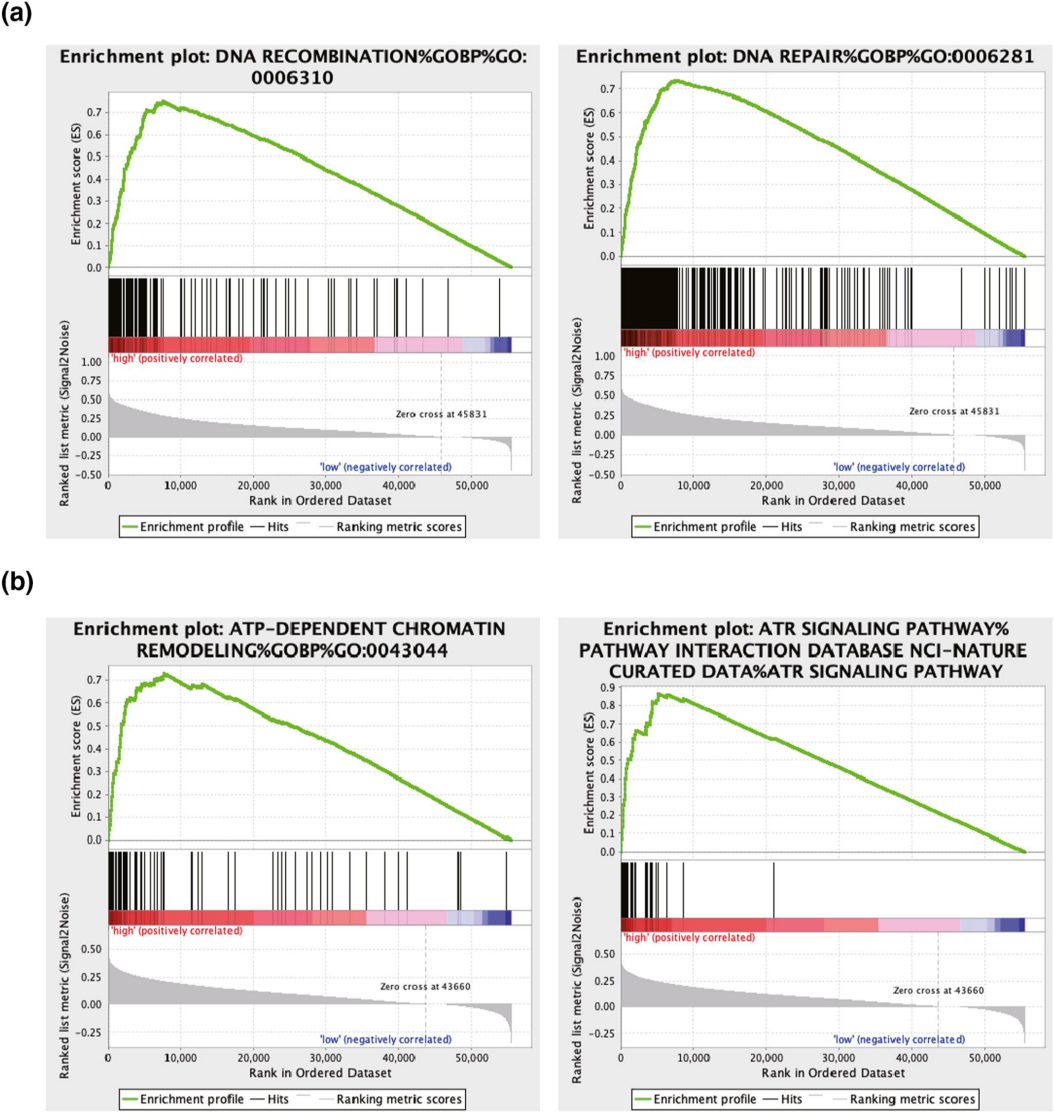
Functional enrichment analysis of YTHDF2 and IGF2BP3. Enrichment results of YTHDF2 (a) and IGF2BP3 (b)

During the RNA methylation process, IGF2BP3 and YTHDF2 act as reader genes. Hence, the roles of m6A dysregulation in the pathogenesis of LIHC were examined here. A pathway enrichment analysis was conducted on samples with various YTHDF2 and IGF2BP3 expression levels. High YTHDF2 expression was associated with the regulation of chromosome organisation, DNA repair, DNA recombination, exonuclease activity, and the negative regulation of DNA metabolic processes (Table S3, Figure 7a). In addition, gene enrichment analysis revealed the association between high IGF2BP3 expression and major biological processes such as double‐strand break repair by means of non‐homologous end‐joining, ATP‐dependent chromatin remodelling, the ATR signalling pathway, and the regulation of chromosome separation (Table S4, Figure 7b). The above results suggested a possible mechanism for the pathogenesis of LIHC.

### 3.7 IGF2BP3 reduction increases HepG2 cell line radiosensitivity in vitro

**FIGURE 8:**
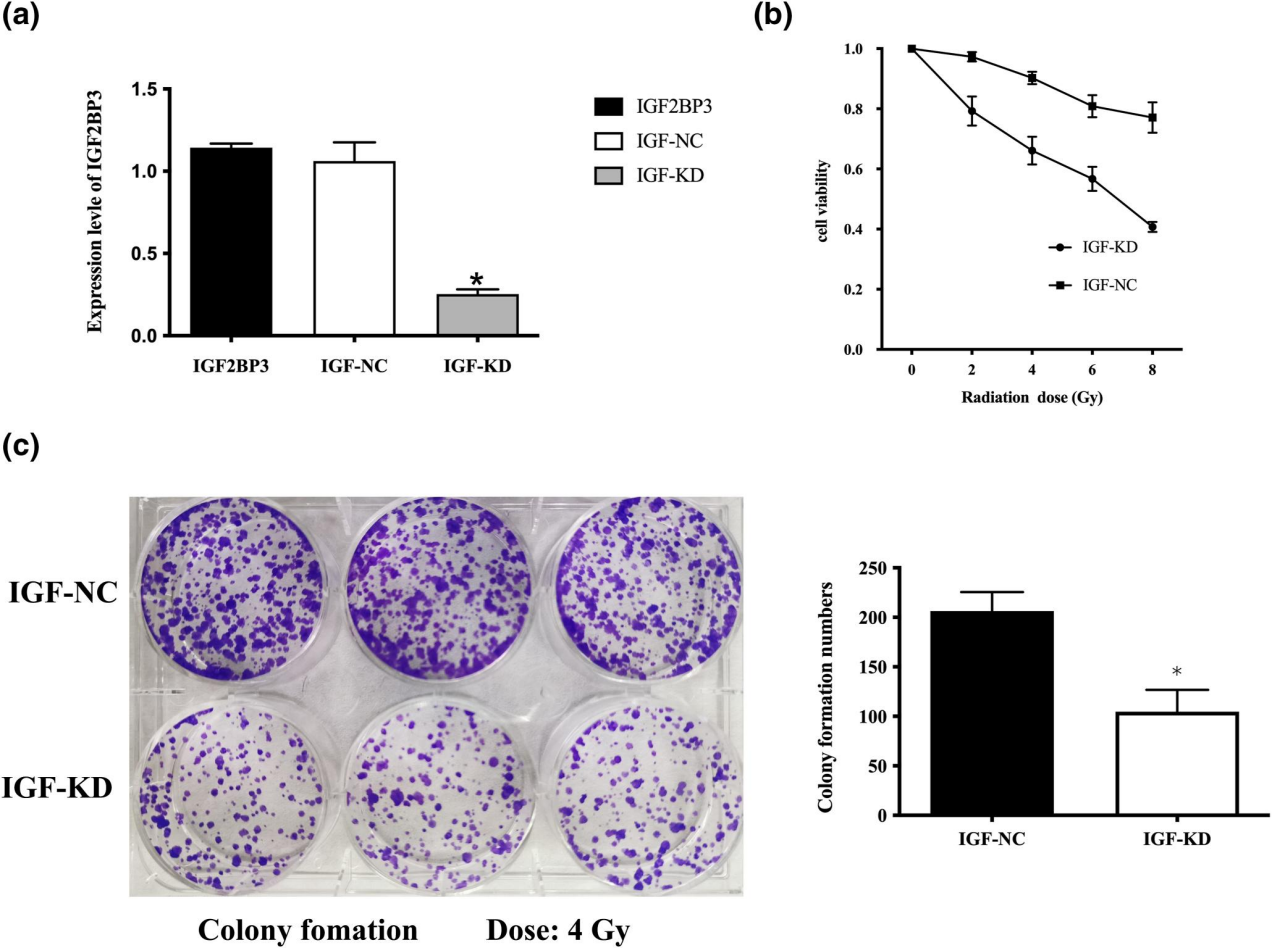
IGF2BP3 reduction increases HepG2 cell line radiosensitivity in vitro. (a) Western blots showed IGF2BP3 expression levels in stable transfected cell lines and the corresponding control cell lines. Histograms show relative western blots expression levels of IGF2BP3 in stable transfected cell lines and the corresponding control cell lines by grayscale analysis. Data are presented as the mean ± _SD. *p < 0.05. (b) Cell proliferation assays show the viability of IGF‐KD after different doses of irradiation as well as the viability of the corresponding control cell lines IGF‐NC. Data are presented as the mean ± _SD (*p*, 0.05). (c) Colony formation assays show the radioresponse of IGF‐KD and the corresponding control cell lines, IGF‐NC. Cells were treated with 4 Gy of irradiation, and the colonies formed after 12 days of incubation were fixed and counted. All experiments were performed in triplicate. IGF2BP3, HepG2 cells without transfection; IGF‐NC, HepG2 cells transfected with empty vector; IGF‐KD, HepG2 cells transfected with IGF2BP3‐knockdown vector

To explore the effect of IGF2BP3 reduction on the LIHC cells’ radiosensitivity, we regulated IGF2BP3 expression by stably transfecting the antisense IGF2BP3 shRNA expression lentivirus into the LIHC cell line HepG2. By doing so, we got a specific cell line with low‐level expression of IGF2BP3 (IGF‐KD) and its corresponding control cell lines (IGF‐NC). Western blot was used to verify the effects of the transfection (Figure 8), and the expression level of IGF2BP3 in IGF‐KD was significantly lower than that of the corresponding control one (Figure 8a) (*p* < 0.05). And then a MTT assay was performed to find out the IGF2BP3 depletion's impact on the HCC cells' viability to irradiation. As shown in the data (Figure 8b), the cell viability rate was apparently lower in the IGF2BP3 low expression cell line under varying degrees of radiotherapy (*p* < 0.05). Furthermore, the radiosensitivity of the cell lines was detected. The colony numbers of HCC cell lines after irradiation were shown in Figure 8c. The results showed that HCC cell lines with down‐regulated IGF2BP3 expression had an obvious drop of colony formation numbers at a 4 Gy irradiation compared to IGF2BP3 positive HCC cell lines (*p* < 0.05). Taken together, the results revealed that IGF2BP3 reduction enhances HCC radiosensitivity in vitro.

### 3.8 IGF2BP3 reduction‐weakened LIHC cells' migration and invasion abilities

**FIGURE 9:**
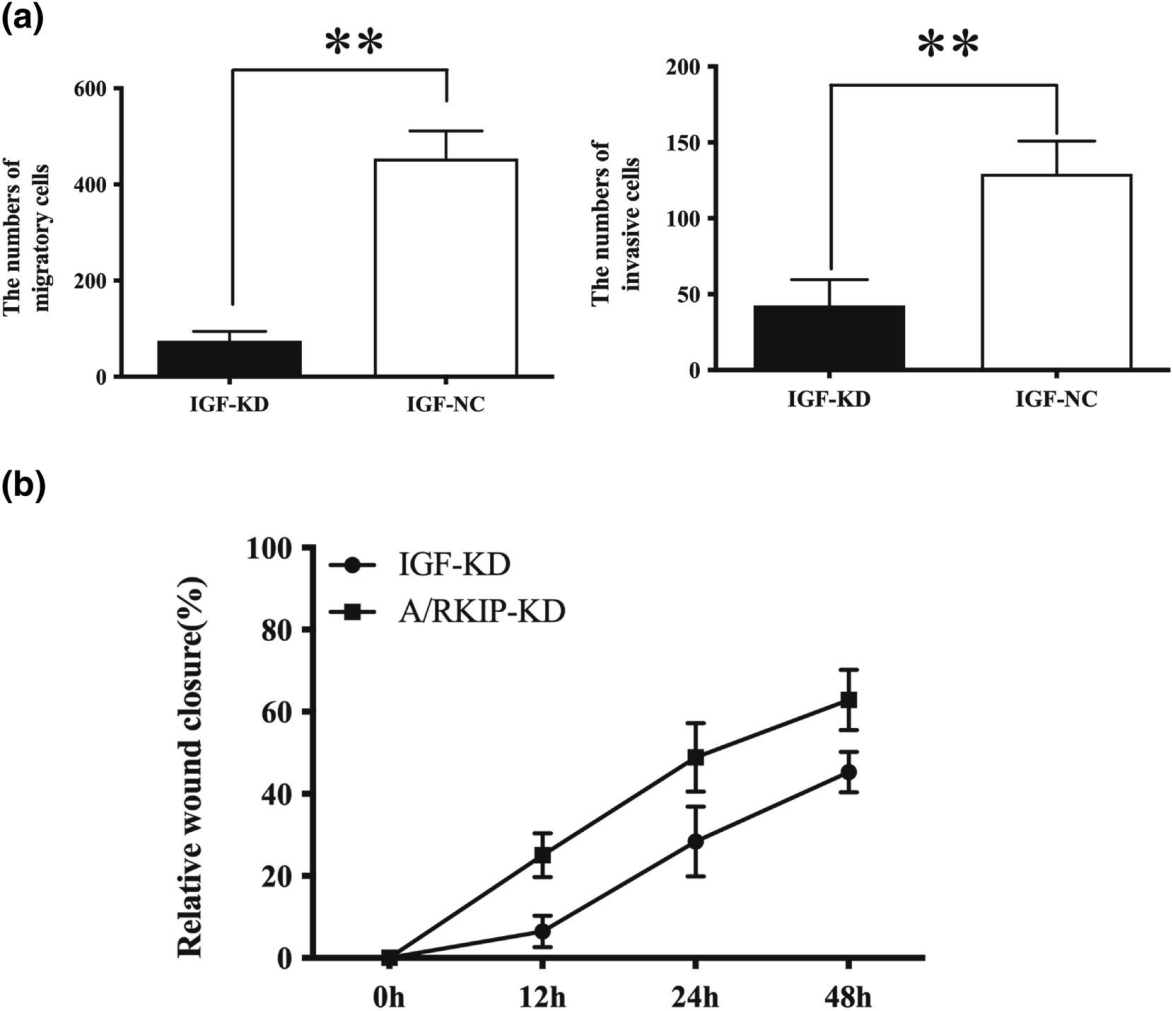
IGF2BP3 reduction weakens liver hepatocellular carcinoma cell migration and invasion abilities. (a) Histograms show numbers of cells of IGF‐KD and its corresponding control cell line IGF‐NC penetrating into the lower chambers. The cells were incubated in the upper chamber for 48 h. **p < 0.01. (b) Histograms show the relative wound closure (present vacant area/original vacant area) of the HCC cell lines in wound‐healing assays at different time points. Data are presented as the mean ± _SD (*p*, 0.05). All experiments were performed in triplicate

Metastatic progression could accelerate in cancer cells when they were under drugs or irradiation; thus metastasis in LIHC would seriously affect the efficiency of chemo and radiotherapy. To explore how IGF2BP3 reduction could affect cell lines' metastatic behaviours, transwell chambers were applied to test cancer cells' migration and invasion abilities. As shown in the data, IGF2BP3 low expression cell lines have weaker migration and invasion abilities (Figure 9a) (*p* < 0.05). Furthermore, a wound healing assay was carried out to discover what influence IGF2BP3 reduction had on cancer cells' metastatic ability. The results revealed that IGF2BP3 expression reduction obviously decreased cells' metastatic abilities (Figure 9b). Overall, IGF2BP3 reduction weakened cell lines' migration and invasion abilities, which means a more satisfactory response to radiotherapy for IGF2BP3 low expression LIHC cells.

## 4 DISCUSSION

Herein, based on the information from LIHC patients, including gene expression, SNV and CNV data from the TCGA database, we performed a comprehensive bioinformatic analysis of gene signatures and prognostic values of m6A regulators in LIHC. Using a univariate Cox regression analysis, we identified 11 m6A regulators in total that had significant correlations with the LIHC prognosis. A 4‐gene signature of m6A regulators was constructed under the support of such models including the LASSO and multivariate Cox regression. It has prognostic value because of the effective prediction of LIHC prognosis. To sum up, genetic alterations in m6A regulatory genes were determined to be reliable and effective biomarkers for predicting the prognosis of LIHC patients.

As various studies have reported, m6A is the most common chemical modification of mRNAs. m6A can control several pathways of gene expression, including processing, degradation and translation [28]. Emerging evidence suggests that m6A is involved in tumour invasion, differentiation, proliferation, and metastasis as an oncogene or a tumour suppressor gene in cancers such as acute myeloid leukaemia, glioblastoma, lung cancer, and breast cancer [6]. For example, the roles of m6A and its methyltransferase METTL3 in osteosarcoma have been reported [29]. It is involved in cell proliferation, migration and invasion. Moreover, METTL3 can regulate the Wnt/β‐catenin signalling pathway and the m6A levels of LEF1, thus promoting osteosarcoma progression. In another study of CRC, METTL3 was reported to impede cell migration and proliferation through the p38/ERK pathway [30]. In gastric cancer, Zhang et al. [31] demonstrated that METTL14 knockdown activated the Wnt and PI3K‐Akt signalling pathways to promote cell invasion and proliferation. The FTO gene as an m6A demethylase was determined to play a crucial role in enhancing melanoma tumourigenesis and anti‐PD‐1 resistance [10]. The knockdown of FTO can increase the sensitivity of melanoma cells to interferon gamma and anti‐PD‐1 therapy.

In hepatocellular carcinoma (HCC), Cheng et al. [32] identified that KIAA1429 can regulate the m6A modification of ID2 mRNA to regulate cell invasion and migration. In another study [33], the reader gene of YTHDF1 experienced significant upregulation in HCC and showed a positive correlation with the pathological tumour stage. The overexpression of YTHDF1 proved to have certain relationships with a poor HCC prognosis.

Based on the microarray and RNA‐seq data from the TCGA and other expression databases, researchers can directly obtain genetic changes in various human cancers. Liu et al. [34] explored the expression of m6A‐related genes with the TCGA, the Human Protein Atlas and the GEO databases. They found the upregulation of more m6A‐associated genes in tumour tissues than in normal tissues, as well as the downregulation of ALKBH5, YTHDF3 and METTL14 in colorectal cancer (CRC). Associations were found between clinical outcomes of CRC patients and the expression levels of ALKBH5, FTO, METTL16, METTL14, and METTL3. In another study of CRC, METTL3 as an oncogene was found to use an m6A‐IGF2BP2‐dependent mechanism to maintain SOX2 expression [35]. In clear cell renal cell carcinoma, Zhou et al. [15] assessed RNA sequencing and CNV data from 528 patients in total from the TCGA database, finding associations between alterations in m6A regulators and the pathologic tumour stage. In this study, genetic alterations in m6A regulatory factors were identified. However, no research on m6A regulatory genes has been conducted with TCGA data in LIHC.

In the functional enrichment analysis of YTHDF2 and IGF2BP3, we found that high YTHDF2 expression was associated with molecular functions related to DNA regulation activities. Defective components in DNA damage and repair mechanisms are the underlying causes of the development and progression of different types of cancer [36]. Additionally, repairing DNA by homologous recombination has proven to be an essential, efficient, high‐fidelity process that repairs DNA damage during cellular metabolism [37]. Compared with normal tissues, both the protein and mRNA levels of YTHDF2 were upregulated in pancreatic cancer tissues [38], and the knockdown of YTHDF2 can increase YAP expression levels and inhibit TGF‐β/Smad signalling. In hepatocellular carcinoma, YTHDF2 directly binds to the m6A modification site of the 3′‐UTR of EGFR to promote the degradation of EGFR mRNA [39]. In addition, high IGF2BP3 expression is associated with the ATR signalling pathway. ATR kinases are key mediators of the DNA damage response that can induce cell cycle arrest and facilitate DNA repair [40]. Highly selective small molecule inhibitors of ATR are now in preclinical and clinical development. IGF2BP3 can be a potential oncogene in gastric carcinogenesis [41]. It has also been identified in various human cancers, such as pancreatic [42], gastric [43], breast [44], and colorectal cancer [45]. In liver cancer, Li et al. [46] proved that its isocorydine derivative can inhibit drug resistance by downregulating IGF2BP3 expression in hepatocellular carcinoma.

Moreover, there are some limitations in our study that should be addressed in future studies. First, this prognostic signature should be verified in large clinical samples. Second, we only determined the biological roles of IGF2BP3 in our study through experiments. For the biological functions of participants in our model, we still need to work hard to explore in future work. Last, there are currently many reports on the prognostic evaluation models of liver cancer. Whether we should compare the predictive performance of these models is worthy of our consideration. To sum up, genetic alterations in m6A regulatory genes were identified as reliable and effective biomarkers for predicting the prognosis of LIHC patients.

## 5 CONCLUSION

Genetic alterations in m6A regulatory genes were determined to be reliable and effective biomarkers for predicting the prognosis of LIHC patients.

## CONFLICT OF INTEREST

The authors declare no potential conflicts of interest in terms of the research, authorship, and/or publication of this manuscript.

## Supporting information

Supplementary Material 1

Figure S1

Figure S2

Figure S3

Table S1

Table S2

Table S3

Table S4

## Data Availability

All data generated or analysed have been included in this study and the supplementary documents.
