# Supplementary material for: A novel m6A‐related prognostic signature for predicting the overall survival of hepatocellular carcinoma patients
Source: IET Syst Biol. 2021 Oct 14;16(1):1–17. doi: 10.1049/syb2.12036 (PMC8849219; doi:10.1049/syb2.12036)
Supplement: Supplementary file 1 — Supplementary Material 1 [file SYB2-16-1-s001.docx]

**Supplementary Information**

**Supplementary Table Legends**

**Supplementary Table 1.** The univariate COX regression analysis of clinical features and m6A regulatory genes.

**Supplementary Table 2.** The univariate COX regression analysis of different m6A regulatory gene expression levels and patients’ prognosis.

**Supplementary Table 3.** Functional enrichment analysis of YTHDF2.

**Supplementary Table 4.** Functional enrichment analysis of IGF2BP3.

**Supplementary Figure Legends**

**Supplementary Figure 1. The expression levels of m6A regulatory genes in different tumor stages.**

**Supplementary Figure 2. Association between CNVs and SNVs of m6A regulatory genes and survival of LIHC patients.** (A) CNV and survival. (B) SNV and survival.

**Supplementary Figure 3. The multivariate COX regression analysis to explore the effects of 17 m6A regulatory genes on the prognosis of patients.** (A) The Kaplan-Meier curves of risk groups and prognosis in LIHC patients. (B) The ROC value based on m6A regulatory genes with 1-year, 3-year, and 5-year survival.
